# Emerging Near-Infrared Targeted Imaging Pharmaceutics for Ovarian Cancer

**DOI:** 10.3390/pharmaceutics18050574

**Published:** 2026-05-06

**Authors:** Angel Phillip, Annu Karithara, Subhash C. Chauhan, Murali M. Yallapu

**Affiliations:** 1 School of Medicine, University of Texas Rio Grande Valley, Edinburg, TX 78539, USA; 2 Division of Cancer and Immunology, Department of Medicine and Oncology, School of Medicine, University of Texas Rio Grande Valley, McAllen, TX 78504, USA

**Keywords:** ovarian cancer, image-guided therapy, fluorophores, mucin, indocyanine green, near-infrared imaging

## Abstract

**Background:** Accurate intraoperative identification of ovarian cancer is challenging, as standard techniques such as visual inspection, palpation, and histopathology often fail to detect microscopic disease. Residual tumor contributes to poor cytoreductive outcomes, high recurrence rates, and chemoresistance. Near-infrared (NIR) imaging using tumor-specific biomarkers has emerged as a promising approach to enhance intraoperative visualization and improve tumor margin delineation. **Methods:** A focused literature review was conducted using PubMed to identify preclinical and clinical studies evaluating NIR image-guided strategies in ovarian cancer. Studies involving tumor-targeted probes against folate receptor alpha, α3-integrin, mesothelin, and CA125 were included, with emphasis on probe design, delivery, imaging performance, safety, and clinical relevance. **Results:** Targeted NIR probes consistently demonstrated improved tumor-to-background contrast, higher lesion detection sensitivity, and enhanced intraoperative guidance compared to conventional imaging. Preclinical and early clinical data indicate favorable safety profiles and minimal off-target toxicity. Evidence suggests that NIR-guided surgery may reduce residual disease burden and potentially improve recurrence-free survival. **Conclusions:** Tumor-specific NIR imaging represents a promising pharmaceutics-based strategy for improving surgical outcomes in ovarian cancer. Despite encouraging results, challenges such as biomarker heterogeneity, limited fluorophore availability, and cost must be addressed. Further large-scale, randomized trials are required to validate efficacy and integrate these approaches into clinical practice.

## 1. Introduction

Ovarian cancer (OC) is a highly gynecological malignancy characterized by the abnormal growth of ovarian epithelial cells. Ovarian cancer is the eighth most common cancer among women worldwide, accounting for about 3% of all female cancers [1]. In the United States alone, an estimated 19,680 new cases are expected. The lifetime risk for a woman developing ovarian cancer is about 1 in 78. Mortality remains high, with about 207,000 deaths reported globally each year [2]. Despite a favorable 5-year survival rate of nearly 90% for stage I disease, most patients are diagnosed at advanced stages, where survival drops to ~30%, largely due to nonspecific early symptoms [3]. The disease predominantly affects postmenopausal women aged 55–65 years, although germ cell and sex cord–stromal tumors are more common in younger individuals.

Ovarian cancer is a highly lethal gynecologic malignancy, with approximately 60% of cases diagnosed at advanced stages due to nonspecific symptoms and lack of effective early screening [4]. Diagnosis relies on histopathologic classification, as ovarian cancer comprises distinct subtypes with different cellular origins, molecular features, and clinical behaviors [5]. Approximately 90% of cases are epithelial ovarian cancers (EOCs), including high-grade serous, low-grade serous, endometrioid, clear cell, and mucinous carcinomas, while germ cell and sex cord–stromal tumors account for the remaining cases [6]. High-grade serous carcinoma is the most prevalent subtype, representing 70–80% of diagnoses, and is typically detected at stage III–IV, where survival remains poor [7]. Epithelial ovarian cancers are further classified into Type I (indolent, genetically stable tumors) and Type II (aggressive, genomically unstable tumors characterized by TP53 mutations), a distinction that informs prognosis and therapeutic strategies [8].

**Figure 1:**
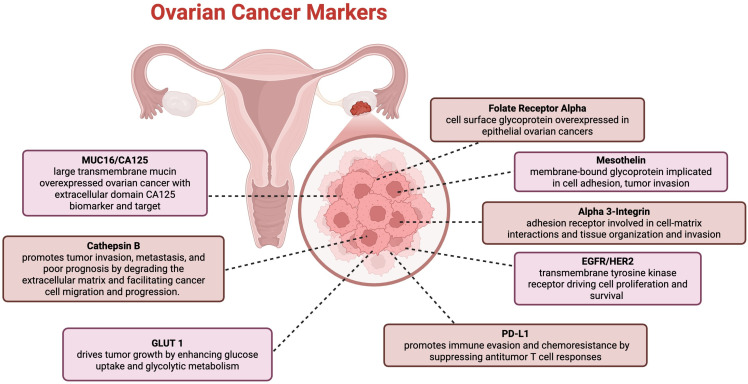
Key molecular targets overexpressed in epithelial ovarian cancer used for tumor-specific NIR fluorescence imaging, including FRα, MUC16/CA125, mesothelin, α3-integrin, EGFR/HER2, PD-L1, cathepsin B, and GLUT1. These biomarkers enable selective probe binding and enhanced intraoperative visualization of malignant tissue. Created in BioRender. Karithara, A. (2026) https://BioRender.com/eew559v (accessed on 29 April 2026).

Screening for ovarian cancer currently relies on a combination of clinical assessment, pelvic examination, serum biomarkers, imaging modalities, and confirmatory histopathology. However, no screening strategy has yet demonstrated sufficient sensitivity and specificity for population-wide early detection, contributing to frequent diagnosis at advanced stages. Biomarkers play a central role in screening and disease monitoring. Cancer antigen 125 (CA125) [9] remains the most widely used serum biomarker, although its sensitivity is limited in early-stage disease and specificity is reduced by benign conditions. Human epididymis protein 4 (HE4) [10,11,12] improves diagnostic accuracy, particularly when combined with CA125. Tumor-associated glycoprotein 72 (CA72-4) [13] offers greater specificity and identifies ovarian cancer cases. Additional biomarkers, including transthyretin (TTR), Cancer antigen 15-3 (CA15-3), glycodelin, and kallikreins, are under investigation. Other markers, macrophage colony-stimulating factor (MCSF), Apolipoprotein A-I (ApoA-I), and inter-alpha-trypsin inhibitor heavy chain H4 (ITIH4) being employed for detection of OC. To enhance performance, multi-marker panels (CA72-4, CA15-3, and MCSF or TTR, ApoA-I, and ITIH4) combining CA125 with complementary proteins have demonstrated improved sensitivity while maintaining high specificity [6,14,15]. Emerging approaches, such as glycomic profiling of CA125, aim to further increase diagnostic precision by exploiting ovarian cancer-specific alterations in glycosylation patterns [16]. Pap smear testing can identify abnormal cervical cells but lacks specificity for cancer, as abnormalities may arise from infection, inflammation, or dysplasia [6]. Elevated platelet counts (400 × 10^9^ U/L) are observed in a substantial proportion of ovarian cancer patients and are associated with advanced disease and poorer survival. Paraneoplastic thrombocytosis, driven by NF-κB and TGFβ/Smad signaling and increased platelet Programmed death-ligand 1 (PD-L1) expression, has shown high sensitivity and specificity for distinguishing ovarian cancer from benign conditions, suggesting potential utility as a low-resource screening approach [17]. Figure 1 illustrates key molecular targets that often overexpress in OC.

The main objective of this review article is to delineate the importance of tumor-specific NIR imaging agents in improving intraoperative lesion detection and cytoreductive completeness in ovarian cancer. This article laid out to review (i) management of ovarian cancer, (ii) imaging modalities of ovarian cancer, (iii) ovarian cancer targets for Near-Infrared imaging, (iv) FDA approved contrast agents and dyes for NIR imaging, (v) limitations and translational challenges of NIR agents in ovarian cancer (Women) health, and (vi) a conclusion section.

## 2. Management of Ovarian Cancer

The management of ovarian cancer requires a multidisciplinary and stage-specific approach that integrates surgical intervention, systemic therapy, and, in select cases, radiation or hormonal treatments. Earlier detection substantially expands therapeutic options and improves clinical outcomes, as multimodal treatments demonstrate the greatest efficacy in early-stage disease. Contemporary management increasingly relies on combining cytoreductive surgery with chemotherapy and targeted therapies, with emerging roles for immunotherapy and hormone-based approaches.

Surgery serves both diagnostic and therapeutic purposes, with the primary goal of achieving maximal cytoreduction, commonly referred to as “debulking,” ideally leaving no macroscopic residual disease. Standard surgical procedures may include total abdominal hysterectomy, bilateral salpingo-oophorectomy, omentectomy, and selective or systematic pelvic and para-aortic lymph node dissection. Given the focus and narrative scope of this review, and owing to space limitations, radiation therapy in the management of ovarian cancer was not addressed; however, commonly employed modalities include external beam radiation therapy, intensity-modulated radiation therapy, and palliative whole-abdominal or localized radiotherapy for symptom control in advanced or recurrent diseases.

### 2.1. Chemotherapy

Primary cytoreductive surgery is typically followed by adjuvant chemotherapy when residual disease is present. In cases where optimal debulking is deemed unachievable at initial presentation, neoadjuvant chemotherapy followed by interval debulking surgery is recommended [18]. The current standard of care consists of maximal surgical cytoreduction combined with platinum- and taxane-based chemotherapy regimens, with carboplatin and cisplatin serving as cornerstone agents. Despite initial responsiveness, a significant proportion of patients develop platinum resistance, necessitating alternative systemic therapies. In such cases, agents such as gemcitabine, liposomal doxorubicin, and bevacizumab are commonly employed. However, treatment efficacy is often limited by cumulative toxicity and adverse effects, leading to premature discontinuation in some patients. The dynamic molecular evolution of OC cells further contributes to chemotherapy resistance, underscoring the need for targeted therapeutic strategies that address both intrinsic and acquired resistance mechanisms.

Targeted therapies have therefore become integral to OC management. Bevacizumab [19], a monoclonal antibody targeting vascular endothelial growth factor (VEGF), inhibits tumor angiogenesis and is frequently used in advanced, recurrent, or persistent OC, often in combination with chemotherapy. A meta-analysis of seven studies demonstrated a significant improvement in progression-free survival among patients with advanced and recurrent disease treated with bevacizumab [20]. Poly (ADP-ribose) polymerase (PARP) inhibitors, including olaparib and niraparib, represent a major advancement in the treatment of BRCA-mutated and homologous recombination-deficient ovarian cancers [21]. These agents exploit defects in DNA repair pathways, leading to genomic instability and cancer cell death [22]. PARP inhibitors are now routinely used as maintenance therapy following chemotherapy and have significantly improved progression-free survival in selected patient populations.

Additional therapeutic modalities include radiation therapy, which is primarily reserved for palliative management or localized metastatic disease. Hormonal therapies, such as tamoxifen and aromatase inhibitors, may be considered in recurrent, estrogen receptor-positive ovarian cancers, particularly in patients who are not candidates for aggressive chemotherapy [23]. Given the high rates of recurrence and multidrug resistance, combination strategies that target OC through multiple biological pathways are essential. Novel drug delivery systems, including nanocarriers, offer promising solutions by bypassing membrane drug efflux pumps, enhancing tumor-specific targeting, and reducing systemic toxicity [24].

### 2.2. Surgical Management

Unlike many solid tumors that form localized masses, ovarian cancer predominantly disseminates through transcoelomic spread along peritoneal surfaces [25]. Malignant cells frequently seed anatomically complex regions such as the mesentery, diaphragm, liver capsule, and pelvic vasculature, complicating complete surgical resection [25]. As a result, extensive cytoreductive procedures may require multiorgan resections, significantly increasing the risk of intraoperative hemorrhage, infection, and postoperative complications, including fistula formation and bowel obstruction [26,27]. Even when optimal cytoreduction is achieved, microscopic residual disease often persists and contributes to disease recurrence, necessitating aggressive postoperative chemotherapy or enrollment in clinical trials involving targeted therapies. Preoperative imaging modalities [28] including computed Tomography (CT), magnetic resonance imaging (MRI), and positron emission tomography (PET) scans frequently underestimate the true extent of peritoneal metastasis, forcing surgeons to rely heavily on intraoperative assessment, which remains inherently subjective and technically challenging. Delayed diagnosis further exacerbates these challenges. Due to nonspecific early symptoms and the absence of effective screening tools, approximately 75% of ovarian cancer cases are diagnosed at advanced stages (III or IV), where surgical morbidity, recurrence rates, and therapeutic resistance are substantially higher [24].

Fluorescence-guided surgery (FGS) has emerged as a promising innovation aimed at improving intraoperative tumor detection and enhancing the completeness of cytoreductive procedures in epithelial ovarian cancer [29]. Traditional surgical techniques based on visual inspection and palpation are limited in their ability to identify microscopic disease, which may harbor chemoresistant cell populations and drive recurrence [30]. FGS addresses this limitation by utilizing fluorescent agents that preferentially accumulate in malignant tissues, enabling real-time visualization of tumors, including submillimeter metastases.

Indocyanine green (ICG), an US Food and Drug Administration (FDA)-approved near-infrared fluorescent dye, is commonly used for lymphatic mapping and tissue perfusion assessment and operates through passive tumor targeting via the enhanced permeability and retention (EPR) effect [20]. In contrast, OTL38 (pafolacianine; Cytalux) is a targeted fluorescent agent that binds to folate receptor-alpha (FRα), which is overexpressed in more than 90% of epithelial ovarian cancers [31]. Clinical studies have demonstrated that OTL38 can detect malignant lesions missed by conventional methods, thereby improving surgical accuracy and potentially patient outcomes. Reported outcomes of FGS are encouraging. Sentinel lymph node detection using ICG demonstrated a mean detection rate of 92.3%, with pelvic and para-aortic detection rates of 94.8% and 96.7%, respectively. During cytoreduction, the sensitivity, specificity, and positive predictive value for micrometastasis detection using OTL38 versus 5-aminolevulinic acid were 92.2% versus 79.8%, 67.3% versus 94.8%, and 55.8% versus 95.8%, respectively [32].

## 3. Imaging

Conventional imaging modalities are central to the diagnosis, staging, therapeutic planning, and post-treatment surveillance of ovarian cancer. An accurate and comprehensive imaging is essential for evaluating disease burden, guiding surgical decision-making, determining resectability, and monitoring treatment response. Transvaginal ultrasound (TVUS) is typically the first-line modality for evaluating adnexal masses. It provides detailed information on the size, structure (solid vs. cystic), presence of septations, and vascular flow patterns, with Doppler studies helping to differentiate between benign and malignant lesions [33]. Despite its utility in characterizing ovarian masses, TVUS is inherently limited in assessing extra-ovarian disease, retroperitoneal lymphadenopathy, and distant metastases, restricting its role in comprehensive staging [34].

CT is the mainstay for preoperative staging and is commonly used to evaluate peritoneal carcinomatosis, omental caking, lymphadenopathy, liver involvement, and ascites. It is also employed post-treatment to assess therapeutic response or detect recurrence [35]. Despite its wide use, CT has reduced sensitivity for detecting sub centimeter peritoneal implants [36]. MRI offers superior soft tissue resolution and is particularly useful when the characterization of an indeterminate pelvic mass is needed [37]. The addition of functional sequences like diffusion-weighted imaging can improve lesion detection [38]. Positron emission tomography combined with CT (PET/CT) using ^18^F-fluorodeoxyglucose (FDG) provides both metabolic and anatomical data [39]. Although not routinely employed for initial diagnosis or staging, FDG PET/CT is particularly useful in the evaluation of suspected recurrent disease, especially in patients with rising CA-125 levels and equivocal findings on conventional imaging [40]. PET/CT also facilitates detection of distant or extra-abdominal metastases and can aid in treatment planning by identifying metabolically active disease sites. Nevertheless, PET/CT is limited by false-positive uptake in inflammatory or benign conditions and reduced sensitivity for low-grade disease conditions. However, despite advances in cross-sectional imaging, none of these modalities reliably detect microscopic residual disease, which remains a major contributor to recurrence. Emerging approaches, including radiomics, machine learning, and artificial intelligence-assisted image analysis, are under active investigation to extract high-dimensional quantitative features from imaging datasets and improve diagnostic precision, prognostication, and treatment stratification.

Intraoperative imaging techniques, particularly FGS, are being explored to overcome the limitations of conventional preoperative imaging. NIR imaging has gained particular attention due to its favorable optical properties, including reduced tissue autofluorescence and improved depth of penetration compared with visible light imaging. Recent advances in NIR imaging within the second near-infrared window (NIR-II; 1000–1700 nm) have further enhanced image quality by enabling deeper tissue penetration and higher signal-to-noise ratios, resulting in improved tumor-to-background contrast [41]. These properties are especially relevant in ovarian cancer, where disseminated peritoneal metastases frequently manifest as small, flat implants on the omentum, bowel serosa, and peritoneal surfaces that are difficult to detect visually or with standard imaging techniques. Preclinical studies have demonstrated the potential of NIR-II imaging for sensitive detection of ovarian cancer metastases. In murine models of advanced-stage disease, NIR-II probes enabled simultaneous real-time visualization of orthotopic primary tumors, regional lymph node involvement, and minute disseminated peritoneal metastases approximately 36 h after systemic administration. Notably, tumor nodules as small as 0.5 mm were detected, outperforming ICG and visible-spectrum fluorophores [42].

**Figure 2:**
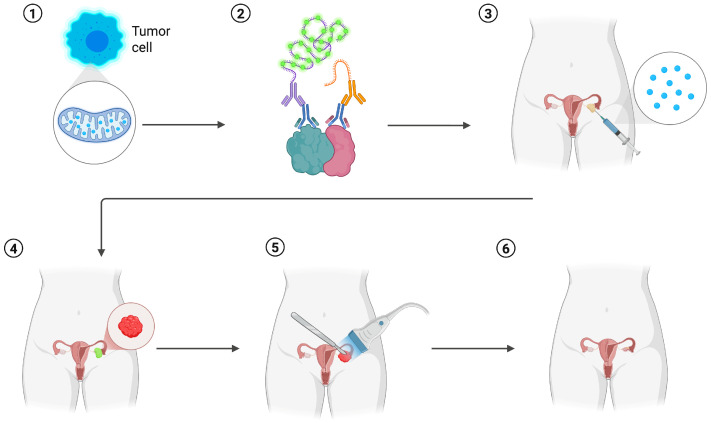
Workflow of targeted NIR imaging in ovarian cancer. Steps include biomarker selection, probe design, systemic probe injection, tumor-specific accumulation, intraoperative NIR imaging, and verification of complete tumor resection. Step 1 to 6 indicates ovarian cancer cells, antibody-dye conjugates specific binding to ovarian cancer cells, targeted binding or targeted delivery of NIR nanoprobes to ovarian tumors, tumor illumination with NIR nanoprobes, surgical imaging or surgical guided removal of tumor glow area (positive tumor margins), and tumor free ovary, respectively. Created in BioRender. Karithara, A. (2026) https://BioRender.com/emm2kyz (accessed on 29 April 2026).

Targeted NIR fluorophores further enhance specificity by exploiting tumor-associated biomarkers. Pafolacianine (OTL38; Cytalux) is a folate receptor-α-targeted NIR agent approved for intraoperative imaging in ovarian cancer [43]. FRα is overexpressed in epithelial ovarian cancers and exhibits limited expression in normal tissues, making it an ideal molecular target [44]. Other investigational approaches include NIR-labeled monoclonal antibodies targeting CA125 (MUC16), a tumor-associated antigen highly expressed in high-grade serous ovarian carcinoma (HGSOC). Preclinical studies have demonstrated enhanced delineation of tumor margins using these targeted probes, highlighting their potential to enable more precise and personalized surgical interventions [45]. While conventional imaging remains foundational to ovarian cancer management, its limited sensitivity for small-volume disease underscores the need for complementary intraoperative technologies. Fluorescence-guided surgery, particularly when combined with NIR and NIR-II imaging and tumor-specific targeting agents, represents a transformative strategy (Figure 2) for improving intraoperative tumor detection, achieving more complete cytoreduction, and ultimately enhancing long-term oncologic outcomes in ovarian cancer patients.

Considering the need for Fluorescence-guided surgery in ovarian cancer management, this review article focuses on providing prime targets for NIR imaging, FDA Approved Contrast Agents and Dyes for NIR Imaging, and limitations and challenges of NIR imaging implementation. At the end a conclusion section is included with overview of NIR imaging and its future directions for developing newer and efficient NIR imaging agents.

## 4. Targets for Near-Infrared (NIR) Imaging

**Scheme 1:**
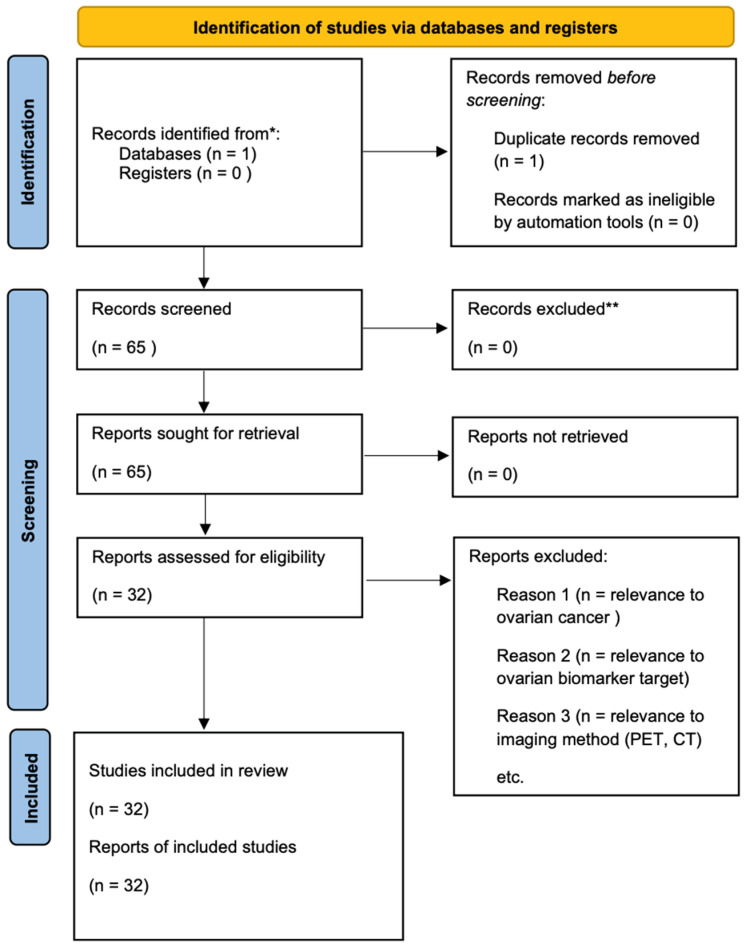
PRISMA diagram for studies selection, eligibility and data extraction. * Indicates the number of records identified from each database or registered searched and ** represents no automation tools used to indicates how many records were excluded from the search.

A comprehensive literature survey was conducted to evaluate the application of NIR image-guided surgical strategies targeting tumor-specific biomarkers in ovarian cancer. Peer-reviewed studies were systematically identified through the PubMed database using structured search queries combining ovarian cancer, near-infrared imaging, and molecular targets of interest in the title/abstract. Scheme 1 represents Preferred Reporting Items for Systematic Reviews and Meta-Analyses (PRISMA) diagram for detailed methodology of literature survey (Scheme 1). Boolean operators were used to combine terms for ovarian cancer, near-infrared imaging, and each molecular target. Only English-language studies in humans and mouse models were included. Articles were then screened for relevance to biomarker-targeted fluorophores, intraoperative imaging performance, and delivery strategies. Studies included both preclinical and clinical trials assessing NIR probe specificity, biodistribution, and cytoreductive accuracy. Primary targets included immune checkpoint and oncogenic markers (PD-L1, EGFR, HER2), lineage- and tumor-associated antigens (FRα, mesothelin, MUC16/CA125), and emerging metabolic or adhesion-related targets (cathepsin B, glucose transporter type 1-GLUT1, and integrin α3). Table 1 provides application of various NIR based image-guided surgical approaches that are widely used in preclinical ovarian cancer studies.

### 4.1. Mesothelin

Mesothelin (MSLN) is a glycosylphosphatidylinositol-anchored cell surface glycoprotein synthesized as a 69 kDa precursor protein that undergoes proteolytic cleavage at arginine 295 (Arg295). This cleavage generates a 31 kDa soluble N-terminal fragment known as megakaryocyte potentiating factor and a 40 kDa membrane-bound C-terminal fragment, mesothelin, which remains anchored to the cell surface. Under physiological conditions, MSLN expression is limited to mesothelial cells lining the pleura, peritoneum, and pericardium; however, it is markedly overexpressed in several malignancies, including ovarian, pancreatic, and mesothelioma tumors [84]. In ovarian cancer, MSLN is overexpressed in approximately 60–70% of high-grade serous carcinomas, making it a highly attractive biomarker for targeted imaging and therapeutic interventions [85]. The interaction between MSLN and CA125 activates downstream oncogenic signaling pathways, including MAPK, NF-κB, and PI3K/AKT, leading to increased tumor cell proliferation, invasion, migration, and resistance to apoptosis. Mesothelin-driven upregulation of matrix metalloproteinases, particularly MMP-7 and MMP-9, further enhances extracellular matrix degradation and metastatic potential [84]. Clinically, circulating mesothelin-related peptides, such as N-ERC/mesothelin, have been investigated as serum biomarkers for early detection and postoperative monitoring in ovarian serous carcinoma, pancreatic adenocarcinoma, and malignant mesothelioma [85,86].

Preclinical studies employing mesothelin-targeted monoclonal antibodies conjugated to NIR fluorophores, such as IRDye800CW, have demonstrated high specificity and favorable tumor-to-background contrast in ovarian cancer xenograft models. For example, anti-MSLN antibody conjugate, i.e., humanized antibody (hYP218) conjugated IR700 showed specific binding to A431/H9 cells both in vitro and in vivo.

These probes enabled real-time intraoperative visualization of both primary tumors and disseminated peritoneal metastases, highlighting their potential utility in fluorescence-guided cytoreductive surgery [87,88]. Beyond full-length antibodies, alternative targeting strategies have been developed to improve tissue penetration and pharmacokinetics. Prantner and colleagues isolated a high-affinity anti- MSLN nanobody (NbG3a) recognizing an epitope within the N-terminal region of mesothelin. This nanobody demonstrated rapid tumor accumulation and clearance from non-target tissues, enabling high-contrast fluorescence imaging and compatibility with multimodal platforms, including MRI [76,89,90]. Mesothelin’s relevance extends beyond imaging into therapeutic innovation. Mesothelin-targeted chimeric antigen receptor (CAR) T-cell and CAR-NK cell therapies have shown potent antitumor activity in both in vitro and in vivo models of MUC16-positive ovarian cancer, reinforcing mesothelin’s role as a multifunctional target for integrated diagnostic and therapeutic (theranostic) applications [91].

Despite its promise, several limitations constrain the widespread application of mesothelin-targeted NIR imaging. Antigen heterogeneity across tumor sites and disease stages can lead to variable probe uptake and false-negative imaging results. Additionally, deep-seated peritoneal metastases may exceed the effective penetration depth of NIR light, limiting signal detection. Endogenous tissue autofluorescence and nonspecific probe accumulation can further reduce signal-to-noise ratios and image contrast. Moreover, “on-target, off-tumor” binding to normal mesothelial tissues raises concerns regarding background signal and potential toxicity, particularly in therapeutic contexts [90,92].

### 4.2. Folate Receptor Alpha

Folate receptor alpha is a glycosylphosphatidylinositol (GPI)-anchored cell surface glycoprotein encoded by the *FOLR1* gene and is characterized by highly restricted expression in normal adult tissues, primarily limited to the apical surfaces of select epithelial cells. In contrast, FRα is frequently and markedly overexpressed in EOCs, particularly in HGSOC, where expression rates exceed 80% in advanced-stage disease [93,94,95]. This tumor-selective expression profile, combined with membrane localization and high ligand affinity, establishes FRα as one of the most clinically validated molecular targets for NIR fluorescence-guided surgery in ovarian cancer.

FRα mediates high-affinity folate uptake through receptor-mediated endocytosis, a process exploited by FRα-targeted imaging agents conjugated to folate analogs or folate-mimetic ligands. NIR fluorophores such as pafolacianine and FolateSiR-1 selectively bind FRα-expressing tumor cells following intravenous administration, leading to preferential accumulation and sustained fluorescence signal within malignant tissues [31,44,46,96,97]. Upon intraoperative excitation, these agents emit NIR fluorescence, enabling real-time, high-contrast visualization of primary tumors and disseminated peritoneal metastases that are frequently occult under conventional white-light inspection.

**Figure 3:**
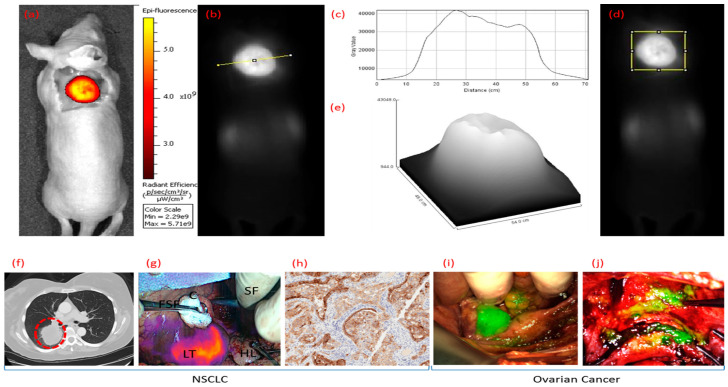
Images illustrate the tumor-to-background ratio of OTL38 using NIR imaging in mouse models and during human surgeries. The images demonstrate clear visualization of (**a**) color and (**b**,**d**) gray scale of mouse bearing FRα-positive tumors after 2 h administration of OTL38. Image J software analysis of tumor measurements of b and d shown in (**c**) and (**e**)**,** respectively. (**f**,**g**) preoperative CT image of pulmonary tumor nodal (shown red circle) and its overlay of fluorescence image over white light image of pulmonary tumor modal, respectively. (**h**) IHC staning of resected pulmonary tumor nodal indicating FRα-positive tumors (**i**,**j**), Fluorescence images over white light images of primary and metastatic overian tumors in uterine adnexa and (**j**) uterus and bladder peritoneum. Note: LT = lung tumor, HL = healthy lung, FSF = Foerster sponge forceps, SF = surgeons fingers, and C = cotton. Reprinted with permission under a Creative Commons Attribution (CC BY) license (http://creativecommons.org/licenses/by/4.0/ (accessed on 29 April 2026)) from ref. [97]. Copyright © 2018 The Authors, published by American Chemical Society, Washington, DC, USA.

OTL38 is a designed for near-infrared imaging, allowing visualization of certain tumors with minimal interference from surrounding tissue (Figure 3) [97]. Its rapid accumulation in folate receptor-positive cancer cells and clearance from non-target tissues enables surgeons to identify malignant lesions intraoperatively using NIR imaging systems.

Beyond imaging, FRα has emerged as a clinically actionable therapeutic target. Antibody–drug conjugates directed against FRα, most notably Mirvetuximab soravtansine-gynx (ELAHERE^®^), have demonstrated clinically meaningful efficacy in patients with platinum-resistant, FRα-high recurrent ovarian cancer. Phase II and III trials reported objective response rates of approximately 34–37%, durable responses, and manageable toxicity profiles, reinforcing the translational relevance of FRα as a unified diagnostic and therapeutic (theranostic) target [98]. The principal clinical advantage of FRα-targeted NIR imaging lies in its capacity to identify additional malignant lesions not detected by visual inspection or palpation, thereby improving the completeness of cytoreductive surgery one of the most robust prognostic factors in ovarian cancer outcomes [31,44,46,50]. Early feasibility studies using EC17, an FRα-targeted fluorescein-based probe, demonstrated the ability of fluorescence guidance to detect otherwise occult disease; however, high background autofluorescence and nonspecific uptake limited its specificity, particularly in non-target tissues [52]. These limitations were subsequently addressed by second-generation NIR agents such as pafolacianine, which operate in the near-infrared spectrum and offer substantially improved tissue penetration and signal-to-noise ratios.

Systematic reviews and meta-analyses have confirmed the added value of FRα-targeted FGCS. Reported true-positive detection rates range from 75–77%, with sensitivities approaching 86%, although lymph nodes remain a recognized source of false-positive signals due to inflammatory uptake or variable receptor expression [53]. Preclinical studies employing dual-modality imaging agents combining fluorescent and nuclear tracers further demonstrated improved tumor-to-blood ratios and enhanced detection of peritoneal metastases, enabling multimodal localization of FRα-expressing lesions [51].

More recent clinical trials using FRα-targeted NIR agents have demonstrated significant improvements in intraoperative detection, with additional lesion identification rates ranging from 29–48% beyond standard inspection. Reported sensitivities exceed 83–97%, with positive predictive values consistently above 88%, reflecting the high tumor specificity of FRα targeting [44,46,50]. Importantly, tumor-to-background ratios achieved with FRα-targeted probes are markedly superior to those of non-targeted fluorophores, owing to minimal FRα expression in healthy tissues and reduced nonspecific uptake [96,99]. Safety profiles across clinical studies have been favorable, with most adverse events being mild, transient, and primarily related to infusion reactions or low-grade gastrointestinal symptoms. No significant dose-limiting toxicities or long-term safety concerns have been reported to date [44,46,50]. Collectively, the medical literature establishes FRα as a clinically validated, tumor-specific biomarker that substantially enhances intraoperative NIR imaging, improves the accuracy and completeness of cytoreductive surgery, and holds promise for integrated theranostic applications in ovarian cancer management.

### 4.3. Alpha 3-Integrin

α3-integrin (α3β1-integrin) is a heterodimeric transmembrane adhesion receptor belonging to the integrin superfamily and is encoded by the *ITGA3* gene. This receptor plays a central role in epithelial cell adhesion, migration, cytoskeletal organization, and wound healing by mediating interactions between cells and extracellular matrix (ECM) components. In normal tissues, α3β1-integrin is expressed at low to moderate levels on epithelial surfaces; however, in invasive and metastatic ovarian carcinomas, its expression is frequently upregulated and functionally associated with enhanced tumor dissemination [100,101,102]. Functionally, α3-integrin activates FAK, Src, and downstream PI3K and MAPK pathways that promote cell survival, migration, and angiogenesis.

Preclinical investigations have explored the utility of α3β1-integrin as a molecular target for NIR imaging. NIR probes conjugated to integrin-binding peptides or monoclonal antibodies have demonstrated selective accumulation in ovarian cancer xenografts and peritoneal metastatic deposits, enabling high tumor-to-background contrast imaging during intraoperative visualization [103]. Furthermore, integrin-targeted nanoplatforms have been engineered to support dual-modality applications, combining NIR fluorescence imaging with therapeutic payload delivery. Such systems leverage α3β1-integrin-mediated internalization to enable targeted drug delivery alongside real-time surgical guidance, positioning α3-integrin as a promising theranostic target [103].

Although much of the early integrin-targeted imaging literature has focused on αvβ3-integrin, these studies have established foundational principles applicable to α3β1-integrin targeting. Using cyclic RGD peptides such as c(KRGDF) conjugated to fluorophores including Cy5.5 and IRDye800, Wang and colleagues successfully visualized integrin-expressing ovarian tumor xenografts. These dual-labeled probes, incorporating both radioisotopes and NIR dyes, enabled complementary nuclear and optical imaging and demonstrated the feasibility of integrin-targeted multimodal detection strategies [83,104]. Importantly, the anatomical proximity of the ovaries to the abdominal surface enhances the effectiveness of NIR imaging, allowing improved detection of integrin-expressing tumor lesions.

Clinical translation of integrin-targeted NIR imaging has yielded encouraging results. In a study evaluating αvβ3-integrin-targeted fluorescence probes during cytoreductive surgery for ovarian cancer, Harlaar et al. [105] reported a sensitivity of 95%, specificity of 88%, and an overall diagnostic accuracy of 96.5%, underscoring the high performance of integrin-based imaging strategies in the intraoperative setting. Moreover, multispectral real-time fluorescence imaging platforms have enabled simultaneous visualization of multiple molecular targets, further enhancing tumor delineation and surgical precision [106].

### 4.4. EGFR and HER2

Epidermal growth factor receptor (EGFR; ERBB1) and human epidermal growth factor receptor 2 (HER2; ERBB2) are transmembrane receptor tyrosine kinases of the ERBB family that regulate cellular proliferation, survival, migration, and differentiation through activation of downstream MAPK/ERK, PI3K/AKT, and JAK/STAT signaling pathways. Dysregulation of these receptors has been implicated in ovarian cancer pathogenesis and progression, making them attractive targets for molecular imaging and targeted therapy.

HER2 is overexpressed in approximately 20–30% of epithelial ovarian cancers, with higher prevalence in mucinous, endometrioid, and clear cell subtypes and relatively low expression in HGSOC. EGFR expressions are more variable across histologic subtypes, and recent molecular profiling studies suggest a largely mutually exclusive expression pattern between EGFR and HER2 in HGSOC, limiting the feasibility of dual-targeted imaging strategies in this subgroup [107,108]. NIR imaging agents targeting HER2 including affibody-based probes, antibody–fluorophore conjugates, and radiolabeled antibodies have demonstrated high sensitivity and specificity for HER2-positive ovarian tumors in both preclinical models and early clinical studies. Systematic analyses of FGCS report sensitivities up to 85.9% and true-positive detection rates of 75–77%, with false-positive rates ranging from 10–25%, largely attributable to lymph node uptake and inflammatory tissue [53].

**Figure 4:**
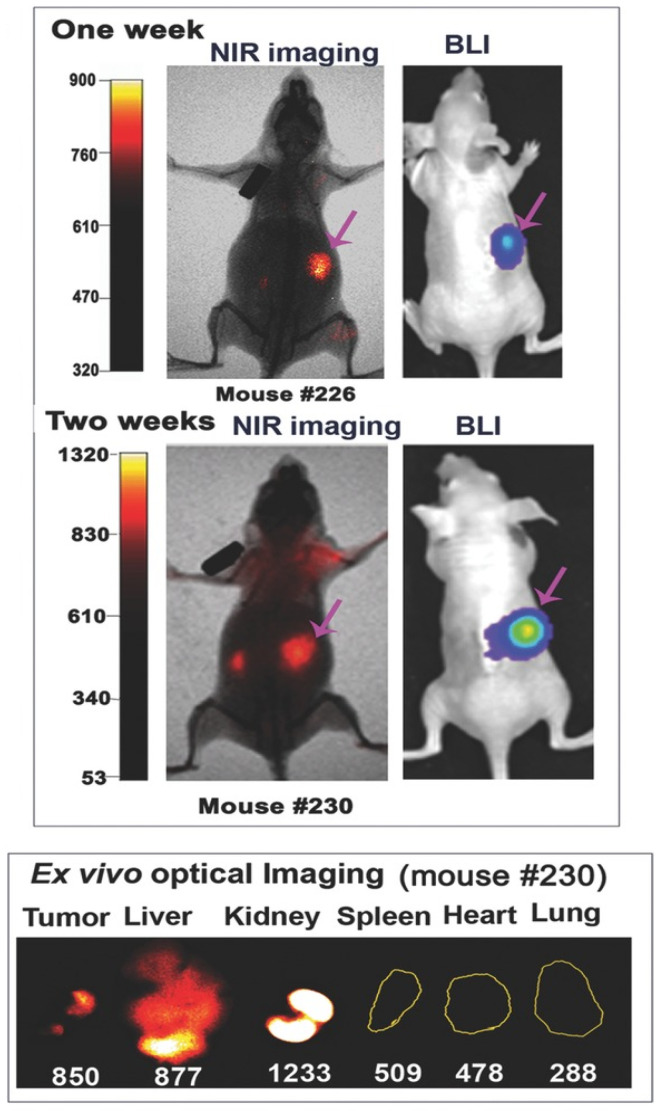
Top image represents NIR optical imaging of early-stage ovarian tumors in mice (one and two weeks) after administration of NIR-830-ZHER2:342–IONPs (red color contrast), showing strong tumor-associated signals confirmed by bioluminescence imaging (blue color contrast). Bottom image demonstrates ex vivo imaging further demonstrate nanoparticle accumulation primarily in tumors (850), with limited distribution in selected organs. Reprinted with permission from [64]. Copyright © 2020 published by American Chemical Society, Washington, DC, USA.

Recent advances in nanotechnology have enabled HER2-targeted nanoprobes capable of dual-modality imaging, including combined photoacoustic and fluorescence tomography for three-dimensional tumor mapping [64,109]. HER2-affibody-conjugated nanoparticles have demonstrated selective tumor accumulation and successful noninvasive MRI/optical imaging of orthotopic ovarian tumors [64] (Figure 4). Additionally, HER2-targeted theranostic nanoparticles delivering cisplatin have shown effective suppression of metastatic disease and image-guided therapy in heterogeneous ovarian cancer models [64,65,67]. Similarly, bioconjugated NIR-II aza-BODIPY dyes linked to trastuzumab have provided deeper tissue imaging and high tumor-to-background ratios for selective visualization of HER2-positive ovarian tumors [54]. EGFR-targeted NIR probes, including cetuximab-Cy7 conjugates and antibody–drug imaging constructs, have demonstrated specific tumor localization in ovarian cancer xenograft models, with optimal imaging windows occurring between 24 and 96 h post-injection. Additional theranostic approaches such as EGFR-specific antibody conjugates with IRDye^®^700 and EGFR-targeted nanoemulsions incorporating platinum prodrugs have enabled precise tumor detection and selective cytotoxicity against EGFR-positive ovarian cancer cells while addressing platinum resistance in preclinical studies [71,110].

While preclinical safety profiles are generally favorable, clinical use of anti-EGFR antibodies is associated with known adverse effects, including dermatologic toxicity, diarrhea, fatigue, and electrolyte disturbances, as reported in randomized therapeutic trials [111]. Non-specific uptake in organs such as the liver, kidney, and bone marrow remains a challenge, emphasizing the need for continued probe optimization to improve tumor-to-background contrast [112]. Overall, EGFR and HER2 represent promising NIR imaging targets in molecularly selected ovarian cancer populations, although broader clinical validation is required.

### 4.5. MUC 16

Cancer antigen 125 (CA125) is a repeating peptide epitope derived from mucin 16 (MUC16), a high-molecular-weight transmembrane mucin extensively expressed in epithelial ovarian cancer. Beyond its diagnostic utility, MUC16 plays an active biological role in tumor progression by promoting cellular proliferation, inhibiting immune surveillance, and facilitating metastatic dissemination. CA125 remains one of the most widely used biomarkers in ovarian cancer for early detection efforts, differential diagnosis of adnexal masses, and longitudinal monitoring of therapeutic response [113]. Recent advances in sequencing, structural modeling, and glycoproteomics have enabled more detailed characterization of MUC16 tandem repeats and glycosylation patterns, improving assay sensitivity and clinical interpretation of CA125 measurements [114]. Since MUC16 promotes peritoneal metastasis through high-affinity interactions with mesothelin, enhancing tumor cell adhesion, increasing matrix metalloproteinase activity, and activating p38 MAPK signaling pathways. These interactions facilitate the formation of three-dimensional multicellular aggregates that disseminate within the peritoneal cavity following detachment from the primary tumor.

**Figure 5:**
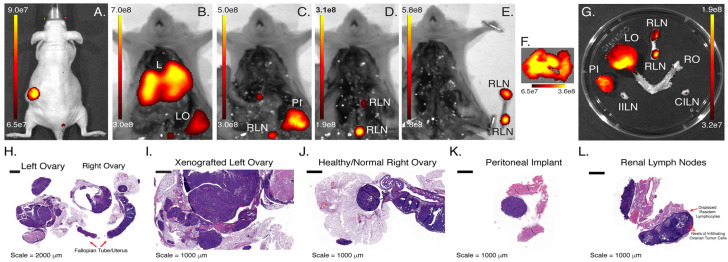
In vivo and ex vivo near-infrared fluorescence (NIRF) imaging with ssB43.13-IR800 in a mouse bearing a CA125-positive OVCAR3 ovarian tumor. The images demonstrate clear detection of (**A**) the primary ovarian tumor (**B**) peritoneal cavity, (**C**) removal of the liver [L] and left ovary [LO], (**D**) the removal of the peritoneal implant [PI], and (**E**) the removal of the renal lymph nodes [RLN], (**F**) the liver and (**G**) the left ovary, right ovary [RO], renal lymph nodes [RLN], ipsilateral inguinal lymph nodes [IILN] and contralateral inguinal lymph nodes [CILN], and (**H**–**L**) confirmation by histopathological analysis of resected tissues. Reprinted with permission from [45]. Copyright © 2020 Wiley-VCH GmbH, Weinheim, Germany.

Radioimmunoscintigraphy and radioimmunotherapy approaches targeting CA125 have demonstrated feasibility for detecting ovarian cancer burden. It was demonstrated in a preclinical study that B43.13-IR800 (a CA125-targeting antibody conjugated to IRDye 800CW) upon site-specific conjugation, it exhibited a superior in vitro binding, in vivo imaging, and validation in human ovarian tumor samples [45] (Figure 5). More recently, antibody–fluorophore conjugates such as AR9.6–IRDye800 have been used for fluorescence-guided resection in pancreatic cancer, highlighting translational potential for ovarian cancer imaging [115]. PET imaging agents based on the B43.13 anti-CA125 antibody have enabled in vivo delineation of primary ovarian tumors and sensitive mapping of metastatic spread to regional lymph nodes, supporting the potential of CA125-directed imaging to improve cytoreductive completeness [45].

### 4.6. Cathepsin B

Cathepsin B is a lysosomal cysteine protease that is frequently overexpressed and aberrantly localized in malignant ovarian tissues, where it contributes to extracellular matrix degradation, tumor invasion, and metastatic progression. Elevated cathepsin B expression correlates with advanced disease stage, poor prognosis, and aggressive tumor behavior in epithelial ovarian carcinoma [116]. In ovarian cancer, patient populations studied include those with epithelial ovarian carcinoma and peritoneal metastases, where cathepsin B levels correlate with disease stage and prognosis [117].

Cathepsin B activated NIR imaging probes are designed as activatable “turn-on” systems that remain optically silent until enzymatic cleavage by cathepsin B within the tumor microenvironment. These probes have demonstrated high sensitivity and specificity in preclinical ovarian cancer models, enabling selective fluorescence activation in malignant tissues while minimizing background signal in normal organs [118]. Detection sensitivity can reach ultralow concentrations (≈0.096 ng/mL), facilitated by probe designs incorporating cathepsin B cleavable peptide substrates and tumor-targeting moieties that enhance retention and imaging window duration [119].

Theranostic applications have further expanded the relevance of cathepsin B targeting. Cathepsin B-responsive doxorubicin prodrug nanoparticles developed for intraperitoneal chemotherapy have demonstrated selective cytotoxicity against cathepsin B-overexpressing ovarian tumors, enhanced intraperitoneal accumulation, and significant inhibition of tumor progression in xenograft and patient-derived models with minimal systemic toxicity [120].

Safety profiles of cathepsin B-activated NIR probes have been favorable in animal studies, with no significant cytotoxicity or off-target organ damage observed following probe administration and laser activation [72]. Although clinical imaging data remain limited, ex vivo studies using human ovarian tumor specimens confirm tumor-specific probe activation, supporting future clinical translation [121]. Overall, cathepsin B-activated NIR imaging offers a highly sensitive and specific approach for visualizing ovarian cancer, with a favorable safety profile in preclinical models.

### 4.7. PD-L1

Programmed death-ligand 1 (PD-L1) is a key immune checkpoint molecule expressed on tumor cells and tumor-associated immune cells, enabling immune evasion through inhibition of cytotoxic T-cell activity. PD-L1 expression has been documented across all major ovarian cancer histologic subtypes, with localization observed in both malignant cells and tumor-associated macrophages [82]. Approximately 47.7% of ovarian cancer samples exhibit high PD-L1 expression, and up to 81% demonstrate tumor-infiltrating lymphocytes expressing CD4 and CD8, underscoring the immunologically active tumor microenvironment [122].

These characteristics have prompted investigation of PD-L1 as a target for NIR photoimmunotherapy (PIT) and fluorescence-guided surgery. Preclinical studies demonstrate that PD-L1-targeted NIR PIT can selectively eradicate ovarian cancer cells, particularly following IFN-γ-induced upregulation of PD-L1 expression [82]. Enhanced uptake observed in some models may be partially attributable to macrophage activation, highlighting the complexity of immune-based imaging.

PD-L1-directed imaging and PIT have demonstrated favorable safety profiles in animal models, with no significant systemic toxicity reported and high survival rates across treated and control cohorts [82,123]. Clinically, the relatively low response rates to PD-L1 inhibition in ovarian cancer may be explained by heterogeneous and often low baseline expression of PD-L1 on tumor cells. Nonetheless, higher PD-L1 expression is associated with poorer prognosis but increased sensitivity to immunotherapeutic strategies, reinforcing the importance of patient selection [124]. With the help of PD-L1, residual tumor and peritoneal metastases can be exposed to NIR light during surgeries and improve treatment outcomes during tumor debulking procedures, filling in an important niche in ovarian cancer treatment in the future [82]. By enabling intraoperative visualization of residual disease and peritoneal metastases, PD-L1-targeted NIR approaches may complement surgical debulking and immunotherapy, filling a critical niche in ovarian cancer management.

### 4.8. GLUT1

Glucose transporter 1 (GLUT1; SLC2A1) is a facilitative transmembrane glucose transporter that is markedly upregulated in malignant ovarian epithelial tumors to support the increased glycolytic demand characteristic of cancer cells. Immunohistochemical analyses consistently demonstrate strong membranous GLUT1 expression in approximately 85–96% of ovarian carcinomas, while expression is minimal or absent in benign and borderline ovarian lesions, highlighting its high specificity for malignancy [125,126,127]. This differential expression profile provides a strong biological rationale for exploiting GLUT1 as a target for molecular imaging.

Leveraging the hyperglycolytic phenotype of ovarian cancer, recent translational studies have developed NIR fluorescence probes that selectively bind GLUT1, enabling sensitive visualization of malignant tissue. These agents demonstrate high tumor-to-background ratios, prolonged tumor retention, and minimal off-target accumulation in preclinical ovarian cancer models, supporting their utility for intraoperative imaging and tumor delineation [128,129]. Investigated patient populations primarily include those with high-grade serous ovarian carcinoma and advanced-stage disease, in which GLUT1 overexpression is associated with aggressive tumor biology, poor clinical prognosis, and reduced likelihood of achieving optimal cytoreduction [130,131].

To date, adverse effects related to GLUT1-targeted NIR imaging agents have not been systematically evaluated in clinical trials; however, available preclinical evidence indicates favorable biocompatibility and low toxicity profiles, with no significant off-target organ damage reported [55,129]. From a diagnostic standpoint, GLUT1 immunohistochemistry exhibits high sensitivity approaching 96% and strong specificity for distinguishing malignant from benign ovarian epithelial tumors [125]. While GLUT1-directed NIR probes consistently demonstrate robust tumor selectivity and contrast in animal models, formal sensitivity and specificity metrics in human ovarian cancer imaging studies remain to be established [130]. Overall, GLUT1 represents a compelling metabolic imaging target in ovarian cancer, supported by strong tissue-based diagnostic performance and growing translational evidence. Continued clinical validation will be essential to define its role in fluorescence-guided surgery and real-time intraoperative tumor detection.

Collectively, EGFR, HER2, MUC16, cathepsin B, PD-L1, and GLUT1 represent complementary molecular targets for near-infrared imaging in ovarian cancer, spanning oncogenic signaling, immune evasion, proteolytic activity, and metabolic reprogramming. Continued optimization of probe specificity, imaging depth (including NIR-II technologies), and patient selection will be critical for translating these approaches into routine clinical practice.

## 5. FDA Approved Contrast Agents and Dyes for NIR Imaging

The U.S. Food and Drug Administration (FDA)-approved and clinically relevant NIR contrast agents are critical for translating fluorescence-guided surgery into routine ovarian cancer care. These agents enable real-time intraoperative visualization of tumor tissue, improving detection of occult and microscopic disease beyond conventional inspection and palpation. Tumor-specific agents, such as folate receptor-targeted tracers, enhance surgical precision by increasing tumor-to-background contrast and supporting optimal cytoreduction, a key determinant of survival. Overall, FDA-approved NIR contrast agents bridge experimental imaging technologies with clinically actionable, patient-centered surgical decision-making in ovarian cancer.

### 5.1. Indocyanine Green

**Figure 6:**
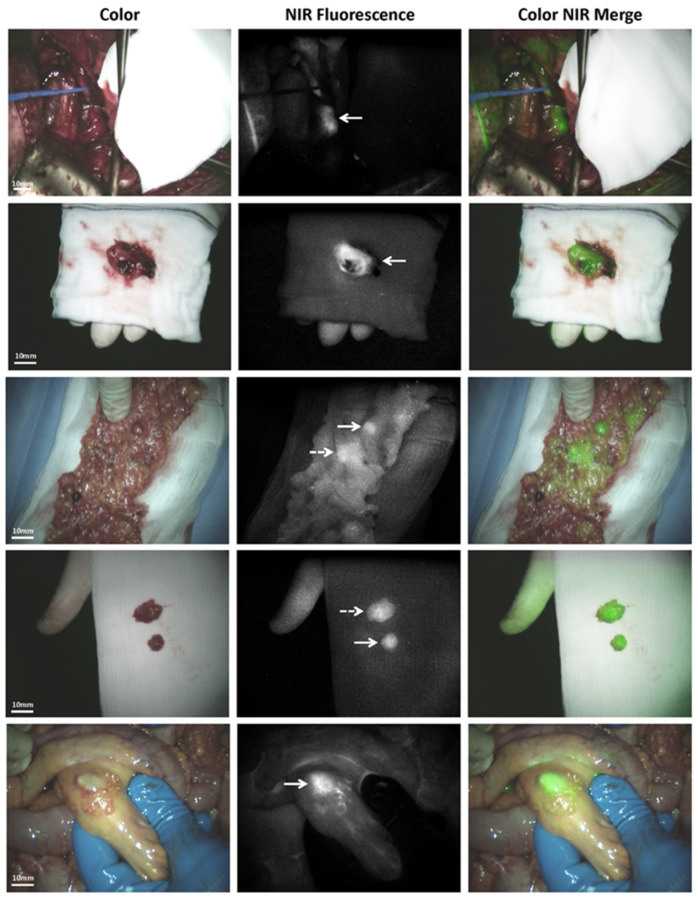
NIR fluorescence imaging detected suspected ovarian cancer lesions in the mesentery, a lymph node near the right iliac vein, and the greater omentum. Ex vivo imaging and histological analysis confirmed metastatic serous adenocarcinoma in most lesions. Arrows point out to the metastases of tumors under NIR fluorescence and that is shown as color NIR merged images. Reprinted with permission under a Creative Commons Attribution (CC BY) license (http://creativecommons.org/licenses/by/4.0/ (accessed on 29 April 2026)) from [133]. Copyright © 2015 The Authors, published by PLOS One, San Francisco, CA, USA.

Indocyanine green is an FDA-approved tricarbocyanine dye that emits fluorescence in the NIR-I spectrum (peak emission ~830 nm) and has been widely adopted for intraoperative visualization of vascular perfusion, lymphatic mapping, and tissue viability across multiple surgical disciplines. In ovarian cancer surgery, ICG is primarily utilized as a non-tumor-specific contrast agent that exploits the EPR effect, whereby macromolecule-bound dyes preferentially accumulate in tumor-associated vasculature due to leaky endothelial junctions and impaired lymphatic drainage [132]. Following intravenous administration, ICG rapidly binds plasma proteins, effectively behaving as a macromolecular tracer that prolongs intravascular residence time while facilitating passive tumor accumulation. Owing to its rapid hepatic clearance and favorable safety profile, ICG is particularly suited for real-time fluorescence imaging during cytoreductive surgery, including visualization of disseminated peritoneal ovarian cancer metastases [133] (Figure 6). Preclinical studies demonstrated that ICG enabled clear delineation of peritoneal metastatic nodules at 6 and 24 h post-injection, achieving significantly higher tumor-to-background ratios compared with non-targeted controls such as IRDye800-albumin, thereby supporting its role in fluorescence-guided endoscopic and open surgical procedures [134].

Clinical data, however, underscore important limitations. In a prospective evaluation of 102 tissue samples, ICG fluorescence demonstrated high sensitivity (91.1%) but very low specificity (13.0%), reflecting a substantial false-positive rate due to non-specific accumulation in inflamed or fibrotic tissues [132]. Despite this limitation, ICG performed particularly well in patients who had received neoadjuvant chemotherapy, where altered tumor vasculature and microenvironment enhanced dye retention, thereby improving detection of residual microscopic disease. Consistently, increased rates of complete cytoreduction were observed in ICG-positive hyperintense regions (46% vs. 30%), suggesting that chemotherapy-induced changes in tumor permeability may augment the utility of EPR-based imaging [135].

Beyond tumor visualization, ICG has gained increasing relevance in sentinel lymph node mapping. NIR fluorescence lymphangiography using ICG enables precise identification of lymphatic drainage pathways and sentinel nodes, potentially reducing the morbidity associated with systematic lymphadenectomy while improving staging accuracy and oncologic safety [37]. Collectively, while ICG lacks tumor specificity and is associated with high false-positive rates, its real-time imaging capability, established safety profile, and utility in vascular and lymphatic mapping continue to support its adjunctive role in ovarian cancer surgery.

### 5.2. Pafolacianine

Pafolacianine (Cytalux^®^) is another FDA-approved, folate receptor alpha (FRα)-targeted NIR fluorescent imaging agent indicated for intraoperative identification of malignant lesions in adult patients with ovarian cancer. The agent consists of a folate analog conjugated to a NIR fluorophore, enabling selective binding to FRα, which is overexpressed in more than 90% of epithelial ovarian cancers while exhibiting minimal expression in normal tissues [31]. Following intravenous administration at the recommended dose of 0.025 mg/kg (1–9 h prior to surgery), pafolacianine accumulates selectively in FRα-positive tumor cells and emits fluorescence upon excitation, allowing real-time intraoperative visualization of malignant tissue using compatible NIR imaging systems. Patients are advised to avoid folate-containing supplements for at least 48 h prior to administration to prevent competitive inhibition of receptor binding [136].

Multiple prospective clinical trials have demonstrated that pafolacianine significantly improves intraoperative lesion detection compared with conventional inspection and palpation alone. Sensitivity rates consistently exceed 80%, and use of the agent has been associated with meaningful increases in complete cytoreduction rates [31,44,46,50]. In a pivotal phase III trial, pafolacianine enabled identification of otherwise occult malignant lesions in 33% of patients, directly altering surgical management and extent of resection [44].

Pafolacianine exhibits favorable optical properties, including a peak emission wavelength of approximately 796 nm, enabling superior tissue penetration and high tumor-to-background contrast relative to visible-light fluorophores [137]. Its ability to illuminate folate receptor-positive lesions intraoperatively has led to its usage for improving surgical precision and outcomes in ovarian cancer patients [138]. Reported adverse events are generally mild and transient, most commonly nausea, vomiting, and abdominal discomfort, with no serious drug-related toxicities observed [44,46,50]. Collectively, pafolacianine represents the most clinically validated tumor-specific NIR imaging agent currently available for ovarian cancer surgery.

### 5.3. Ga-68-Trivehexin

Gallium-68 Trivehexin is an investigational positron emission tomography (PET) radiotracer targeting integrin αvβ6, a cell-surface receptor highly upregulated in multiple epithelial malignancies, including ovarian carcinoma. Although not FDA approved, ^68^Ga-Trivehexin is undergoing early-phase clinical evaluation as a molecular imaging agent for improved disease mapping and preoperative staging. Initial clinical studies demonstrate that ^68^Ga-Trivehexin PET/CT provides superior tumor-to-background contrast compared with standard ^18^F-FDG PET/CT, particularly within the abdomen, owing to minimal physiological uptake in normal tissues. This feature enables more accurate visualization of peritoneal disease burden, which is frequently underestimated by conventional imaging modalities in advanced ovarian cancer [139].

The radiotracer is synthesized with high radiochemical purity (>95%) and demonstrates favorable pharmacokinetics, including rapid clearance from non-target tissues and intense uptake in αvβ6-expressing tumors [114,140]. Early clinical experience suggests that 68Ga-Trivehexin may improve preoperative staging and disease mapping, potentially informing surgical planning and therapeutic decision-making in ovarian cancer [141]. While initial studies have focused on safety, biodistribution, and imaging performance, there is currently no established dosing regimen for routine clinical use, and its role in patient management remains investigational. The broader literature on gallium-68 radiopharmaceuticals highlights the rapid expansion of targeted PET imaging agents in oncology, with 68Ga-Trivehexin representing a promising addition for molecular imaging of integrin αvβ6-positive malignancies [141,142,143]. Further prospective trials are needed to validate diagnostic accuracy, clinical impact, and potential theranostic applications in ovarian cancer.

## 6. Limitations and Translational Challenges

While NIR imaging agent potential is evident, its clinical adoption is still limited by several factors that warrant careful consideration. Traditional imaging modalities like CT and MRI are essential for preoperative staging, but they lack the sensitivity to capture the extent of deeper peritoneal disease that must the address intraoperatively which NIR can directly address. However, the challenge of inherent marker variability in ovarian cancer can lead to false negative results, or cells may not successfully accumulate in the fluorescent probe. Moreover, the high cost of specialized imaging equipment and regulatory obstacles for approving novel fluorophores can also slow the implementation of these techniques into standard oncological practices. The deep-seated nature of certain peritoneal metastases may be a challenge for NIR light penetration, the endogenous autofluorescence may occasionally interfere with the signal’s clarity and potentially increase false positive in non-malignant or inflammatory tissues.

To fully understand the clinical application of NIR fluorescent guided imaging and surgery, several directs will need to be explored. The most critical aspect to be evaluated is the execution of large-scale, randomized clinical trials to conclusively validate the preliminary findings and establish standardized protocols to definitively confirm the improvement of recurrence free survival outcomes from ovarian cancer. Next, integrating artificial intelligence and machine learning to analyze NIR image data can enhance detection precision and provide surgeons with stronger reliable objective guidance. The development of new fluorophores with enhanced visual properties and integration of multiomic biomarker profiling can also help tailor treatment to each patient. Thus, combining NIR with therapeutic modalities like photodynamic therapy can be a potential and promising theranostic approach in treating residual microscopic diseases after cytoreduction.

Among the reviewed targets, FRα currently shows the strongest translational readiness due to its combination of high tumor specificity, favorable safety, and prospective clinical validation with pafolacianine. In contrast, ICG remains clinically accessible but lacks tumor specificity and demonstrates substantial false-positive signaling, limiting its standalone value for precise lesion discrimination. Mesothelin, CA125, integrin-based probes, HER2, and EGFR each show promising preclinical or early translational utility, but their broader adoption is limited by variable target expression, off-target uptake, and the relative scarcity of ovarian cancer-specific clinical trials. Emerging targets such as cathepsin B, PD-L1, and GLUT1 are compelling for theranostic or activatable imaging strategies, however they remain in earlier stages of development and lack robust ovarian cancer clinical validation. Those that combine high tumor specificity, low background signal, established dosing feasibility, and compatibility with real-time cytoreductive workflows are of high use in therapy.

## 7. Conclusions

Near-infrared fluorescence imaging is reshaping the intraoperative management of ovarian cancer by enabling precise, real-time visualization of tumor deposits during cytoreductive surgery. Compared with conventional inspection and palpation, NIR-guided approaches improve detection of occult and microscopic peritoneal disease, supporting more complete cytoreduction, the strongest predictor of survival. Targeted agents, particularly FRα-directed tracers such as pafolacianine, have demonstrated meaningful improvements in lesion detection, while advances in fluorophore design, including NIR-II imaging (1000–1700 nm), offer enhanced tissue penetration, reduced autofluorescence, and superior contrast, further expanding clinical potential [132,133,134]. Clinically targeted NIR agents show strong diagnostic performance for microscopic disease detection, with intravenous delivery remaining the most effective route for systemic distribution. Emerging nanoparticle-based platforms incorporating receptor-targeted and pH-responsive designs further improve tumor accumulation, signal stability, and imaging duration [133,135,136]. In contrast, non-targeted agents continue to face limitations due to higher false-positive rates, underscoring the importance of tumor-specific targeting.

Despite its promise, clinical translation is limited by regulatory challenges, lack of standardized imaging protocols, cost, and the need for long-term outcome validation. Tumor heterogeneity, variable biomarker expression, and interpatient pharmacokinetics may also impact imaging reliability. Additionally, technical barriers such as limited penetration depth, photobleaching, and dependence on specialized equipment must be addressed. Fluorescence-guided surgery also presents challenges, including false negatives due to inconsistent tracer uptake and false positives from non-specific accumulation. Regulatory hurdles and the cost of targeted agents such as OTL38 further restrict widespread adoption [137].

Future efforts should focus on large-scale randomized trials to determine survival benefits, along with the development of standardized protocols and FDA-approved tumor-specific fluorophores. Integration with artificial intelligence, multimodal imaging, and theranostic strategies, such as combining NIR imaging with photodynamic or photoimmunotherapy, represents a promising direction for improving detection and treatment of residual disease.

## Figures and Tables

**Table 1:** Preclinical and clinical application of NIR image-guided surgical strategies for ovarian cancer.

Title of the Article and Citation	Target Agent, Study Design, and Animal Model/Clinical Trial Information	Key Findings or Outcome of the Study
Folate receptor-alpha		
† A phase II, multicenter, open-label trial of OTL38 injection for the intra-operative imaging of folate receptor-alpha positive ovarian cancer, Randall et al., [46]	OTL38, a folate–indole–cyanine green-like conjugate targeting FRα, was evaluated in a Phase II, multicenter, open-label, prospective human clinical trial.	OTL38-based NIR imaging demonstrated a favorable safety and efficacy profile, achieving sensitivity of up to 97.97% and a positive predictive value of 94.93%, with 48.3% of patients having at least one additional malignant lesion detected exclusively by OTL38 and only mild drug-related adverse events reported.
Comparison of Five Near-Infrared Fluorescent Folate Conjugates in an Ovarian Cancer Model, García de Jalón et al., [47]	Five folate-conjugated NIR fluorophores, ZW800-1, ZW800-1 Forte, IRDye^®^ 800CW, ICG-OSu, and an in-house Cy7 derivatives were evaluated in a comparative preclinical study using a subcutaneous SKOV-3 ovarian cancer xenograft mouse model, with systematic assessment of probe performance in both in vitro and in vivo settings.	The choice of fluorophore markedly influences biodistribution and tumor-to-background contrast, with ZW800-1 Forte demonstrating the highest tumor-specific contrast and specificity among the evaluated agents.
High Spatiotemporal Near-Infrared II Fluorescence Lifetime Imaging for Quantitative Detection of Clinical Tumor Margins, Chen et al., [48]	A folate receptor-targeted indocyanine green nanoprobe was evaluated in a comparative study using the SK-OV-3 ovarian cancer xenograft model, with systematic assessment of targeting and imaging performance across in vitro, in vivo (mouse), and ex vivo (human ovarian tumor tissue) platforms.	FL-tumor imaging using the folate receptor-targeted ICG nanoprobe enabled high-contrast, quantitative tumor margin delineation with superior accuracy (90%) and consistency compared with conventional NIR-II fluorescence imaging (58%), achieving a signal-to-background ratio of 10.8 and demonstrating highly specific targeting of folate receptor alpha.
† A Phase III Study of Pafolacianine Injection (OTL38) for Intraoperative Imaging of Folate Receptor–Positive Ovarian Cancer (Study 006) Tanyi et al., [44]	OTL38 was evaluated in a Phase III, open-label, multicenter (11 centers) confirmatory human clinical trial involving patients with known or suspected ovarian cancer scheduled for cytoreductive surgery; participants received 0.025 mg/kg IV pafolacianine at least one hour before surgery, and intraoperative NIR imaging was employed to detect malignant lesions.	In 33% of patients (95% CI, 24.3–42.7%) on tissue not planned for resection and undetectable by white light or palpation, exceeding the prespecified 10% threshold; ovarian cancer detection sensitivity was 83%, with a patient-level false-positive rate of 24.8%, and complete R0 cytoreduction was achieved in 62.4% of patients, while drug-related adverse events occurred in 30% (predominantly mild: nausea, vomiting, abdominal pain) with no serious drug-related events or deaths reported.
Membrane cholesterol enrichment and folic acid functionalization lead to increased accumulation of erythrocyte-derived optical nano-constructs within the ovarian intraperitoneal tumor implants in mice, Lee et al., [49]	Erythrocyte-derived nano-constructs encapsulating ICG, enhanced with membrane cholesterol and functionalized with folic acid to target folate receptor-α, were evaluated in a preclinical in vivo study using a mouse model with intraperitoneal ovarian tumor implants, enabling comparative assessment of tumor targeting and imaging performance.	Nano-constructs combining cholesterol enrichment and folic acid functionalization demonstrated approximately six-fold higher tumor accumulation than free ICG, two-fold greater uptake than cholesterol-only constructs, and 33% higher accumulation compared to FA-only constructs at 24 h post-injection, highlighting their enhanced efficacy for imaging ovarian tumors.
† Enhancing surgical precision in ovarian cancer with FRα-fluorescence-guided surgery, Ferrari et al., [50]	A systematic review encompassing 11 clinical and preclinical studies evaluated the safety, efficacy, and feasibility of folate receptor alpha-targeted fluorescence imaging in ovarian cancer surgery using agents such as OTL38 and EC-17 disodium salt (EC17), without introducing new animal models.	OTL38 markedly enhanced intraoperative detection of malignant lesions, identifying an additional 33% of tumors with sensitivity exceeding 85%, while EC17 proved feasible but was limited by tissue autofluorescence; most adverse events were mild, and overall, FRα-targeted imaging contributed to improved completeness of cytoreductive surgery.
Improved Intraoperative Detection of Ovarian Cancer by Folate Receptor Alpha Targeted Dual-Modality Imaging, Hekman et al., [51]	In a preclinical intraperitoneal ovarian cancer mouse model, this study evaluated ^111^In-farletuzumab-IRDye800CW for dual-modality imaging, with biodistribution assessed three days post-injection and FRα specificity confirmed by blocking studies and immunohistochemistry.	This study demonstrated that dual-modality imaging enabled specific visualization of intra-abdominal ovarian tumor lesions with minimal normal tissue uptake, allowing real-time fluorescence-guided resection. Competitive blocking with unlabeled farletuzumab confirmed FRα specificity, and the approach achieved favorable tumor-to-blood ratios (3.4–3.7), supporting its feasibility for intraoperative detection of ovarian cancer metastases.
† Intraoperative imaging of folate receptor alpha positive ovarian and breast cancer using the tumor specific agent EC17, Tummer et al., [52].	In a prospective clinical study, patients with FRα-positive ovarian and breast cancer received intravenous EC17 prior to surgery, and intraoperative fluorescence imaging was performed to evaluate tumor detection, specificity, and sensitivity in correlation with histopathology and FRα expression, alongside safety and pharmacokinetic assessment.	In ovarian cancer patients, intraoperative EC17 fluorescence imaging identified 57 lesions, including 16% of malignant lesions missed by conventional inspection, with fluorescence correlating with FRα expression; however, high tissue autofluorescence limited specificity, particularly in breast cancer, despite an overall favorable safety profile.
The feasibility of folate receptor alpha- and HER2-targeted intraoperative fluorescence-guided cytoreductive surgery in women with epithelial ovarian cancer: A systematic review, de Jong et al., [53]	A systematic review of fluorescence-guided cytoreductive surgery (FGCS) evaluated FRα-targeted agents (e.g., OTL38, EC17) and HER2-targeted probes in both animal models of epithelial ovarian cancer (EOC) and human EOC patients, assessing their safety, tumor specificity, intraoperative detection efficacy, and impact on completeness of cytoreduction.	FGCS targeting FRα or HER2 is feasible in preclinical and clinical settings, improving intraoperative tumor detection with sensitivities up to 85.9% and true-positive rates of 75–77%. While false positives (10–25%), mainly from lymph nodes, remain a limitation, these approaches show strong potential to enhance cytoreduction, warranting further validation of survival benefit.
† A Novel Tumor-Specific Agent for Intraoperative Near-Infrared Fluorescence Imaging: A Translational Study in Healthy Volunteers and Patients with Ovarian Cancer, Hoogstins et al., [31]	OTL38 was evaluated in a translational Phase I/II study, demonstrating favorable safety and pharmacokinetics in healthy volunteers and ovarian cancer patients.	Intraoperative NIR imaging with OTL38 enabled detection of an additional 29% of malignant lesions not identified by inspection or palpation, with selective accumulation in FRα-positive tumors, supporting its feasibility and clinical utility for tumor-specific surgical guidance.
Human epidermal growth factor receptor 2		
NIR-II Aza-BODIPY Dyes Bioconjugated to Monoclonal Antibody Trastuzumab for Selective Imaging of HER2-Positive Ovarian Cancer, Godard et al., [54]	NIR-II aza-BODIPY dyes bioconjugated to trastuzumab were evaluated in a preclinical SKOV-3 HER2-positive ovarian cancer xenograft mouse model.	The bioconjugated NIR-II dyes demonstrated prolonged serum stability and preserved HER2-binding affinity, enabling selective, high-contrast in vivo imaging of HER2-positive ovarian tumors, thereby supporting their potential application in fluorescence-guided surgical resection.
Moderating hypoxia and promoting immunogenic photodynamic therapy by HER-2 nanobody conjugate nanoparticles for ovarian cancer treatment, Zhang et al., [55]	HER2-targeted nanobody-conjugated human serum albumin nanoparticles loaded with chlorin e6 (Ce6) and catalase (Nb@HCC) tested in HER2-positive SK-OV-3 ovarian tumors, enabling targeted photodynamic therapy.	Nb@HCC nanoparticles, when combined with NIR irradiation, effectively alleviated tumor hypoxia, downregulated HIF-1α expression, and induced immunogenic cell death, while promoting dendritic cell maturation; additionally, they synergized with anti-CTLA-4 therapy to suppress distant tumor growth, achieving potent antitumor effects with minimal systemic toxicity.
Virus-resembling nano-structures for near infrared fluorescence imaging of ovarian cancer HER2 receptors, Guerrero et al., [56]	Genome-depleted brome mosaic virus (BMV) nanoparticles, doped with ICG and covalently functionalized with anti-HER2 monoclonal antibodies, utilized to verify specific binding to HER2-expressing ovarian cancer cell lines in vitro, supporting their potential use as targeted NIR imaging agents for HER2-positive tumors.	Virus-mimicking nanostructures effectively targeted HER2-overexpressing ovarian cancer cells and enabled high-contrast NIR fluorescence imaging in vitro, highlighting their potential as precise, receptor-specific imaging platforms.
Functionalized polymeric nanoparticles loaded with indocyanine green as theranostic materials for targeted molecular near infrared fluorescence imaging and photothermal destruction of ovarian cancer cells, Bahmani et al., [57]	Anti-HER2 antibody-functionalized, PEGylated polymeric nanoparticles loaded with ICG tested for selective binding with HER2-high SKOV3 cells over HER2-low OVCAR3 cells, enabling targeted NIR fluorescence imaging and photothermal tumor cell ablation.	Functionalized nanoparticles demonstrate enhanced specificity for HER2-overexpressing ovarian cancer cells, provided superior NIR fluorescence imaging, and achieved markedly higher photothermal-mediated cell destruction compared to free ICG and non-targeted nanoparticle controls.
Optical Imaging of Ovarian Cancer Using HER-2 Affibody Conjugated Nanoparticles, Satpathy et al., [58]	This study aimed to develop and evaluate HER2-targeted iron oxide nanoparticles conjugated with an NIR dye for specific in vivo imaging of orthotopic ovarian cancer xenografts, integrating nanoparticle synthesis, tumor model establishment, and multimodal imaging to assess targeting efficiency and biodistribution.	HER-2 affibody-conjugated nanoparticles selectively accumulated in HER-2 positive ovarian tumors, enabling sensitive and specific noninvasive detection of both primary and disseminated tumors in the peritoneal cavity; multimodal imaging with a single agent was feasible.
Fluorescence Lifetime Imaging for Quantification of Targeted Drug Delivery in Varying Tumor Microenvironments, Verma et al., [59]	This study is about evaluating and quantifying the binding and distribution of the HER2-targeted monoclonal antibody trastuzumab (TZM) using NIR fluorescence resonance energy transfer (FRET) imaging in HER2-positive breast and ovarian cancer cell lines and xenograft mouse models, with validation by immunohistochemistry.	NIR FLI–FRET imaging quantitatively assessed trastuzumab–HER2 binding in vitro and in vivo, revealing lower antibody binding in SKOV-3 ovarian tumors than in breast cancer xenografts due to stromal and vascular barriers, and establishing FLI as a robust method to monitor antibody delivery and target engagement.
Near Infrared Photoimmunotherapy in the Treatment of Disseminated Peritoneal Ovarian Cancer, Sato et al., [60]	This investigation is aimed at evaluating the therapeutic potential of a trastuzumab-IRDye700DX antibody-photosensitizer conjugate (tra-IR700) for photoimmunotherapy (PIT) in HER2-positive ovarian cancer, including its cytotoxic effects in vitro (cell culture and 3D spheroids) and its efficacy in treating disseminated peritoneal and flank xenograft tumors in mouse models.	NIR-PIT induced light dose-dependent cytotoxicity in vitro and complete tumor cell killing in 3D spheroids; in vivo, NIR-PIT significantly reduced tumor volume and bioluminescence in both peritoneal and flank models compared to controls, demonstrating effective cell killing in HER2-positive disseminated ovarian cancer.
Virus-Mimicking Nanoparticles for Targeted Near Infrared Fluorescence Imaging of Intraperitoneal Ovarian Tumors in Mice, Vankayala et al., [61]	This study is aimed at evaluating the tumor-targeting efficiency and imaging performance of virus-mimicking nanoparticles (optical viral ghosts, OVGs) functionalized with anti-HER2 antibodies in a mouse model of intraperitoneal ovarian cancer, comparing them to non-functionalized OVGs and free dye using quantitative NIR fluorescence imaging.	Functionalized ovarian-targeting vesicles (OVGs) produced markedly higher tumor fluorescence and signal-to-noise ratios than non-functionalized controls and free dye, enabling enhanced tumor visualization at 24 h post-injection.
Improved Debulking of Peritoneal Tumor Implants by Near-Infrared Fluorescent Nanobody Image Guidance in an Experimental Mouse Model, Debie et al., [62]	This study evaluates the feasibility and efficacy of fluorescence-guided surgery using an IRDye800CW-labeled anti-HER2 nanobody in an intraperitoneal ovarian cancer mouse model, comparing tumor detection and resection outcomes against conventional surgical methods.	Fluorescence-guided surgery enabled clear visualization of submillimeter tumor nodules, significantly reduced residual tumor burden, increased sensitivity from 59.3% to 99.0%, and decreased false positive rate from 19.6% to 7.1% compared to conventional surgery.
Human epidermal growth factor receptor-2 antibodies enhance the specificity and anticancer activity of light-sensitive doxorubicin-labeled liposomes, Li et al., [63]	It examines the specificity, tumor-targeted accumulation, and therapeutic efficacy of HER2 antibody-conjugated liposomes containing doxorubicin and hollow gold nanospheres, using a dual HER2-positive and HER2-negative tumor mouse model with NIR irradiation.	HER2 antibody-conjugated liposomes showed >2-fold higher accumulation in HER2-positive tumors and, upon NIR irradiation, achieved up to 92.7% tumor growth inhibition without systemic toxicity through combined photothermal and chemotherapeutic effects.
Active Targeting Using HER-2-Affibody-Conjugated Nanoparticles Enabled Sensitive and Specific Imaging of Orthotopic HER-2 Positive Ovarian Tumors, Satpathy et al., [64]	This study aimed to investigate the targeting efficiency, biodistribution, and NIR imaging capability of HER2 affibody-conjugated magnetic iron oxide nanoparticles in an orthotopic ovarian cancer mouse model, comparing HER2-high and HER2-low tumor xenografts.	HER2-targeted iron oxide nanoparticles enabled highly sensitive dual-modality optical/MRI detection of ovarian tumors as small as 1 mm, with tumor uptake strictly dependent on HER2 overexpression.
Targeted Drug Delivery and Image-Guided Therapy of Heterogeneous Ovarian Cancer Using HER2-Targeted Theranostic Nanoparticles, Satpathy et al., [65]	It proposed to study the theranostic potential of amphiphilic polymer-coated magnetic iron oxide nanoparticles conjugated with NIR-labeled HER2 affibody and cisplatin, focusing on targeted drug delivery, imaging-guided therapy, and therapeutic efficacy in heterogeneous HER2-expressing orthotopic ovarian cancer xenografts.	HER2-targeted nanoparticles selectively delivered cisplatin to primary and metastatic tumors, with imaging signals correlating with HER2 expression and therapeutic response. Long retention of nanoparticles enabled detection of resistant residual tumors by molecular imaging.
Intraoperative Near-Infrared Fluorescence Tumor Imaging with Vascular Endothelial Growth Factor and Human Epidermal Growth Factor Receptor 2 Targeting Antibodies, Scheltinga et al., [66]	This study focused on evaluating bevacizumab (anti-VEGF) and trastuzumab (anti-HER2) conjugated to IRDye 800CW, with ^89Zr-labeled counterparts for PET imaging, in mice bearing subcutaneous and intraperitoneal ovarian cancer xenografts, assessing tumor uptake, biodistribution, and intraoperative imaging performance.	NIR fluorescence-labeled bevacizumab and trastuzumab enabled highly sensitive and specific in vivo detection of VEGF- and HER2-positive tumor lesions, including submillimeter deposits. Tumor-to-background ratios reached 1.93 ± 0.40 for bevacizumab and 2.92 ± 0.29 for trastuzumab at six days post-injection, with findings validated by histopathology, immunohistochemistry, and fluorescence microscopy
HER-2/neu targeted delivery of a nanoprobe enables dual photoacoustic and fluorescence tomography of ovarian cancer, Xi et al., [67]	This study investigates a HER-2/neu-targeted nanoprobe in an orthotopic human ovarian cancer xenograft mouse model, focusing on targeted delivery and dual-modality imaging performance in vivo.	HER-2/neu-targeted bevacizumab and trastuzumab enabled highly sensitive and specific in vivo detection of VEGF- and HER2-positive tumor lesions, including submillimeter deposits. Tumor-to-background ratios reached 1.93 ± 0.40 for bevacizumab and 2.92 ± 0.29 for trastuzumab at six days post-injection, with findings validated by histopathology, immunohistochemistry, and fluorescence microscopy
Epidermal growth factor receptor		
Near-infrared fluorescent molecular probes with cetuximab in the in vivo fluorescence imaging for epithelial ovarian cancer, Zhang et al., [68]	This investigation explores Cetuximab-Cy7 in a subcutaneous SKOV3-Luc ovarian cancer xenograft mouse model, assessing nanoprobe development, targeted tumor uptake, distribution patterns, and imaging quantification.	Cetuximab-Cy7 selectively targeted EGFR-positive ovarian cancer xenografts, providing strong tumor-specific fluorescence with optimal imaging between 24 and 96 h post-injection.
NIR-II Navigation with an EGFR-Targeted Probe Improves Imaging Resolution and Sensitivity of Detecting Micrometastases in Esophageal Squamous Cell Carcinoma Xenograft Models, Wang et al., [69]	This investigation examines cetuximab-IR800 in esophageal squamous cell carcinoma mouse xenograft models, comparing the performance of NIR-I and NIR-II imaging for accurate tumor localization, detection of metastatic lymph nodes, and intraoperative surgical guidance.	In EGFR-positive esophageal cancer models, cetuximab-IR800 combined with NIR-II imaging significantly improved tumor contrast and enabled detection of subcentimeter metastases with enhanced spatial resolution.
The effect of subcellular localization on the efficiency of EGFR-targeted VHH photosensitizer conjugates, Lith et al., [70].	This study shows how VHH 7D12 and VHH-CPP nanobodies conjugated to IRDye700DX are helpful to target EGFR-positive cancer cell lines and patient-derived ovarian cancer ascites cells, assessing how surface-bound versus internalized conjugates mediate phototoxicity upon light activation in vitro.	EGFR-targeted nanobody photosensitizer conjugates induced selective phototoxicity in EGFR-expressing cells, with superior efficacy observed for membrane-bound compared to internalized constructs.
Detection and Specific Elimination of EGFR+ Ovarian Cancer Cells Using a Near Infrared Photoimmunotheranostic Approach, Bauerschlag et al., [71]	It proposes the site-specific conjugation of IRDye700 to the EGFR-targeting scFv-425-SNAP-tag in EGFR-positive ovarian cancer cell lines and patient-derived tissues/ascites, evaluating NIR imaging capabilities and light-induced cytotoxicity in vitro and ex vivo.	These findings were further supported by studies demonstrating highly specific detection and elimination of EGFR-positive ovarian cancer cells and patient-derived ascites cells, with IC_50_ values in the low nanomolar range, establishing a framework for precise EGFR-directed theranostic conjugates.
Cathepsin B		
In vivo near-infrared imaging and phototherapy of tumors using a cathepsin B-activated fluorescent probe, Chen et al., [72]	It aims to assess a Cathepsin B-activated fluorescent probe (CyA-P-CyB) in mouse tumor xenograft models, evaluating its specificity, near-infrared imaging performance, and phototherapeutic efficacy in both in vitro and in vivo settings.	Cathepsin B-responsive probes enabled tumor-specific NIR fluorescence activation and phototoxicity, with minimal off-target signal and no detectable toxicity in major organs.
Optical Imaging of Cancer-Related Proteases Using Near-Infrared Fluorescence Matrix Metalloproteinase-Sensitive and Cathepsin B-Sensitive Probes, Yhee et al., [73]	This study evaluates role of a Cathepsin B-sensitive polymer nanoparticle probe in mouse tumor xenograft models via enzyme-responsive activation approach. This study reports tumor-targeted imaging, and comparative performance in both in vitro and in vivo settings.	These probes exhibited cytoplasmic fluorescence recovery following enzymatic cleavage, supporting their utility for evaluating cytoplasmic drug delivery.
A folate receptor-specific activatable probe for near-infrared fluorescence imaging of ovarian cancer, Lee et al., [74]	The objective of this study is to identify a folate receptor-targeted, Cathepsin B-activatable probe in mouse ovarian cancer xenograft models, assessing its selective in vivo activation, tumor-specific near-infrared imaging, and potential for real-time lesion visualization.	Fluorescence activation occurred selectively after cathepsin B-mediated cleavage in folate receptor-positive cancer cells, further enhancing tumor specificity.
Glucose Transporter 1		
Expression of GLUT-1 glucose transfer, cellular proliferation activity and grade of tumor correlate with [F-18]-fluorodeoxyglucose uptake by positron emission tomography in epithelial tumors of the ovary, Kurokawa et al., [75]	This study assesses imaging capabilities of [^18^F]-FDG PET in ovarian cancer patients, focusing on GLUT1-mediated tracer uptake for tumor detection, metabolic activity assessment, and correlation with clinical and pathological features.	FDG uptake (SUV) strongly correlates with GLUT1 expression and tumor grade, noninvasive biomarker for ovarian cancer.
Mesothelin		
Molecular Imaging of Mesothelin-Expressing Ovarian Cancer with a Human and Mouse Cross-Reactive Nanobody, Prantner et al., [76]	The nanobody NbG3a potential as NIRF and MRI agent. It uses subcutaneous A1847 xenografts and syngeneic orthotopic ID8 ovarian cancer models, to assess in vivo imaging performance, biodistribution, and epitope specificity.	Rapid, specific tumor accumulation; detectable at 4 h, persists to 48 h; diffuse intratumoral distribution.
Rapid nanobody-based imaging of mesothelin expressing malignancies compatible with blocking therapeutic antibodies, Benloucif et al., [77]	This study utilizes an anti-mesothelin nanobody toward mesothelin-positive tumor xenografts, focusing on in vivo PET imaging performance and tracer localization.	Demonstrated same-day imaging capability and good target specificity.
CA125 (Mucin 16)		
Engineering of a Fully Human Anti-MUC-16 Antibody and Evaluation as a PET Imaging Agent, Babeker et al., [78]	To study outcomes of a fully human monoclonal antibody M16Ab, DFO-conjugated and ^89^Zr-labeled, in OVCAR3, SKOV3, and SW1990 xenograft models, assessing in vivo PET imaging over 24–120 h post-injection and ex vivo biodistribution.	Specific uptake in MUC16-expressing tumors; retention up to 120 h; high tumor-to-blood ratios; minimal uptake in negative controls (SKOV3).
A Molecularly Targeted Intraoperative Near-Infrared Fluorescence Imaging Agent for High-Grade Serous Ovarian Cancer, Fung et al., [45]	A preclinical study to examine B43.13-IR800, a CA125-targeting antibody conjugated to IRDye 800CW, in subcutaneous, orthotopic, and patient-derived xenograft models of high-grade serous ovarian cancer, focusing on site-specific conjugation, in vitro binding, in vivo imaging, and validation using human tumor samples.	B43.13-IR800 specifically binds CA125-expressing ovarian cancer cells and enables sensitive NIRF imaging of HGSOC tumors in mouse models; probe also binds and identifies CA125-expressing cells in human primary tumor and metastatic lymph node samples.
PD-L1		
Imaging PD-L1 Expression with ImmunoPET, Truillet et al., [79]	To evaluate the role of ^89^Zr-labeled human IgG1 antibody C4 for tumor uptake and biodistribution using PET/CT in xenograft models.	^89^Zr-C4 detected low levels of PD-L1 in tumors; strong specificity; could detect changes in post treatment.
Anti-Human PD-L1 Nanobody for Immuno-PET Imaging: Validation of a Conjugation Strategy for Clinical Translation, Bridoux et al., [80]	Ga-68-labeled PD-L1 nanobody testing for in vivo tumor targeting and biodistribution in xenograft models.	Specific tumor uptake (~1.8%IA/g), good targeting, renal clearance, stable in vivo.
Immuno-PET Imaging of Tumour PD-L1 Expression in Glioblastoma, Sharma et al., [81]	ImmunoPET imaging capacity of novel ^18^F-AlF- and ^68^Ga-labeled Z_PD-L1 affibody, and evaluating its biodistribution, and correlation with PD-L1 expression in subcutaneous and orthotopic GBM xenograft models.	Could discriminate high vs. low PD-L1 expression, fast clearance, high contrast even at 1 h post injection.
PD-L1 near Infrared Photoimmunotherapy of Ovarian Cancer Model, Jin et al., [82]	The application of an anti-PD-L1 antibody–dye conjugate in a syngeneic orthotopic ovarian cancer mouse model, assessing its performance for in vivo NIR imaging, photoimmunotherapy, and real-time tumor visualization.	The PD-L1 probe localized tumors; imaging guided therapeutic effect in ovarian cancer model.
α3 Integrin		
Near-Infrared Optical Imaging of Ovarian Cancer Xenografts with Novel α3-Integrin Binding Peptide “OA02”, Aina et al., [83]	The fate of α3-integrin-targeting peptide “OA02” labeled with Cy5.5 or AlexaFluor in nude mice bearing ES-2 (α3-positive) and Raji (α3-negative) xenografts, evaluating in vivo NIR imaging specificity and blocking effects.	Rapid, selective tumor uptake within 15 min; lasted up to ~70 min for some probes; binding blocked by anti-α3 antibody.

† Indicates clinical trial or clinical studies.

## Data Availability

No new data was created.

## Abbreviations

The following abbreviations are used in this manuscript:

ApoA-I	Apolipoprotein A-I
CA15-3	Cancer antigen 15-3
CA72-4	Tumor-associated glycoprotein 72
CA125	Cancer antigen 125
CT	Computed Tomography
EC17	EC-17 disodium salt
EGFR	Epidermal growth factor receptor
EOC	Epithelial ovarian cancer
EPR	Enhanced permeability and retention
FDA	Food and Drug Administration
FDG	^18^F-fluorodeoxyglucose
FGS	Fluorescence-guided surgery
FRα	folate receptor-alpha
GLUT1	Glucose transporter type 1
HER2	Human epidermal growth factor receptor 2
HE4	Human epididymis protein 4
HGSOC	High-grade serous ovarian carcinomas
IRDye 800CW	IRDye 800CW Carboxylate
ITIH4	Inter-alpha-trypsin inhibitor heavy chain H4
MUC16	Cancer antigen 125
MCSF	Macrophage colony-stimulating factor
MRI	Magnetic resonance imaging
MSLN	Mesothelin
NIR	Near-infrared
OC	Ovarian cancer
OTL 38	Pafolacianine
PARP	Poly (ADP-ribose) polymerase
PD-L1	Programmed death-ligand 1
PET	Positron emission tomography
TTR	Transthyretin
TVUS	Transvaginal ultrasound
VEGF	Vascular endothelial growth factor
